# Symmetry-Breaking-Induced Frequency Combs in Graphene Resonators

**DOI:** 10.1021/acs.nanolett.2c00360

**Published:** 2022-07-29

**Authors:** Ata Keşkekler, Hadi Arjmandi-Tash, Peter G. Steeneken, Farbod Alijani

**Affiliations:** † Department of Precision and Microsystems Engineering, Delft University of Technology, Mekelweg 2, Delft 2628 CD, The Netherlands; ‡ Kavli Institute of Nanoscience, Delft University of Technology, Lorentzweg 1, Delft 2628 CJ, The Netherlands

**Keywords:** Nanoelectromechanical systems (NEMS), graphene, nonlinear dynamics, internal resonance, frequency combs

## Abstract

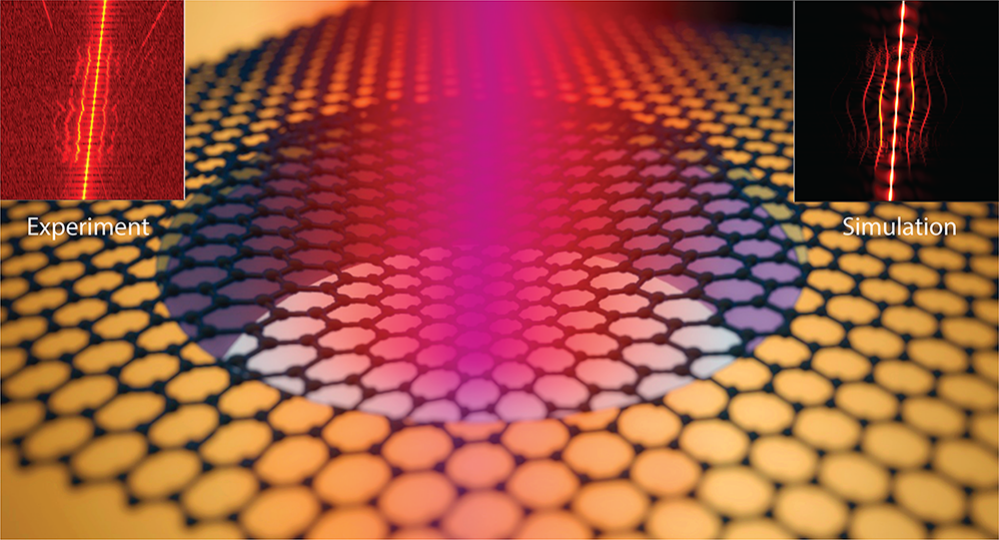

Nonlinearities are inherent to the dynamics of two-dimensional materials. Phenomena-like intermodal coupling already arise at amplitudes of only a few nanometers, and a range of unexplored effects still awaits to be harnessed. Here, we demonstrate a route for generating mechanical frequency combs in graphene resonators undergoing symmetry-breaking forces. We use electrostatic force to break the membrane’s out-of-plane symmetry and tune its resonance frequency toward a one-to-two internal resonance, thus achieving strong coupling between two of its mechanical modes. When increasing the drive level, we observe splitting of the fundamental resonance peak, followed by the emergence of a frequency comb regime. We attribute the observed physics to a nonsymmetric restoring potential and show that the frequency comb regime is mediated by Neimark bifurcation of the periodic solution. These results demonstrate that mechanical frequency combs and chaotic dynamics in 2D material resonators can emerge near internal resonances due to symmetry-breaking.

Nanomechanical resonators made of two-dimensional (2D) materials are ideal for exploring nonlinear dynamic phenomena. Because of their atomic thickness and high flexibility, forces in the piconewton range can already trigger large-amplitude oscillations in these membranes and drive them into nonlinear regime.^1,2^ Tension modulation via electrostatic actuation^3−5^ and opto-thermal forces^6,7^ serve as practical knobs to tune mechanical nonlinearity of 2D material membranes and can lead to a wealth of nonlinear phenomena including multistability,^8^ parametric resonance,^6,9^ parametric amplification,^10,11^ high-frequency tuning,^12,13^ stochastic switching,^14^ and mode coupling.^15,16^

Among different nonlinear phenomena that emerge in 2D material membranes, mode coupling is particularly interesting as it allows for the transfer of energy between vibrational states of single^15^ or coupled 2D resonators.^17^ Mode coupling is also closely linked to nonlinear dissipation^9,18^ and can be tuned utilizing internal resonance (IR), a condition at which two or more resonance frequencies become commensurate. The application of IR in mechanical resonators spans from frequency division^19^ and time-keeping^20,21^ to enhancing the sensitivity of scanning probe techniques.^22^

Here, we present a mechanism for generating frequency combs by symmetry breaking that exploits internal resonances of a few nm-thick graphene resonator. We make use of the extreme flexibility of graphene to controllably break its out-of-plane symmetry by bending it using electrostatic force and achieve a one-to-two (1:2) IR between its distinct mechanical modes. Unlike recent demonstrations of frequency comb generation in graphene that require strong coupling of the suspended membrane with a high quality factor SiN_*x*_ substrate,^23^ here we show that by careful tuning of the intermodal coupling between two modes of vibration in a single resonator, frequency combs can be generated. As a result of this 1:2 modal interaction, we observe splitting of the resonance peak at a critical gate voltage and drive level, leading to equally spaced spectral lines near the fundamental resonance. By using an analytical model that accounts for the broken symmetry and comprises quadratic coupling terms, we account for the characteristic dependence of the frequency comb region on the membrane tension and deflection amplitude and confirm that symmetry-broken mechanics lies at the root of the observations.

Experiments are performed on a 15 nm thick exfoliated graphene flake and transferred over a circular cavity of 8 μm in diameter and of 220 nm depth forming a drum resonator. The motion of graphene is read-out in a Fabry-Pérot interferometer where a red helium–neon laser (λ = 633 nm) is used to probe the motion^24,25^ (see Figure 1a,c). The drum is driven opto-thermally using a power modulated blue laser (λ = 485 nm), and to control the static deflection of the drum a local gate electrode is placed at the bottom of the cavity, see Figure 1b. Moreover, to reduce damping by the surrounding gas, the sample is measured in a vacuum chamber with pressure ≤10^–4^ mbar.

**Figure 1:**
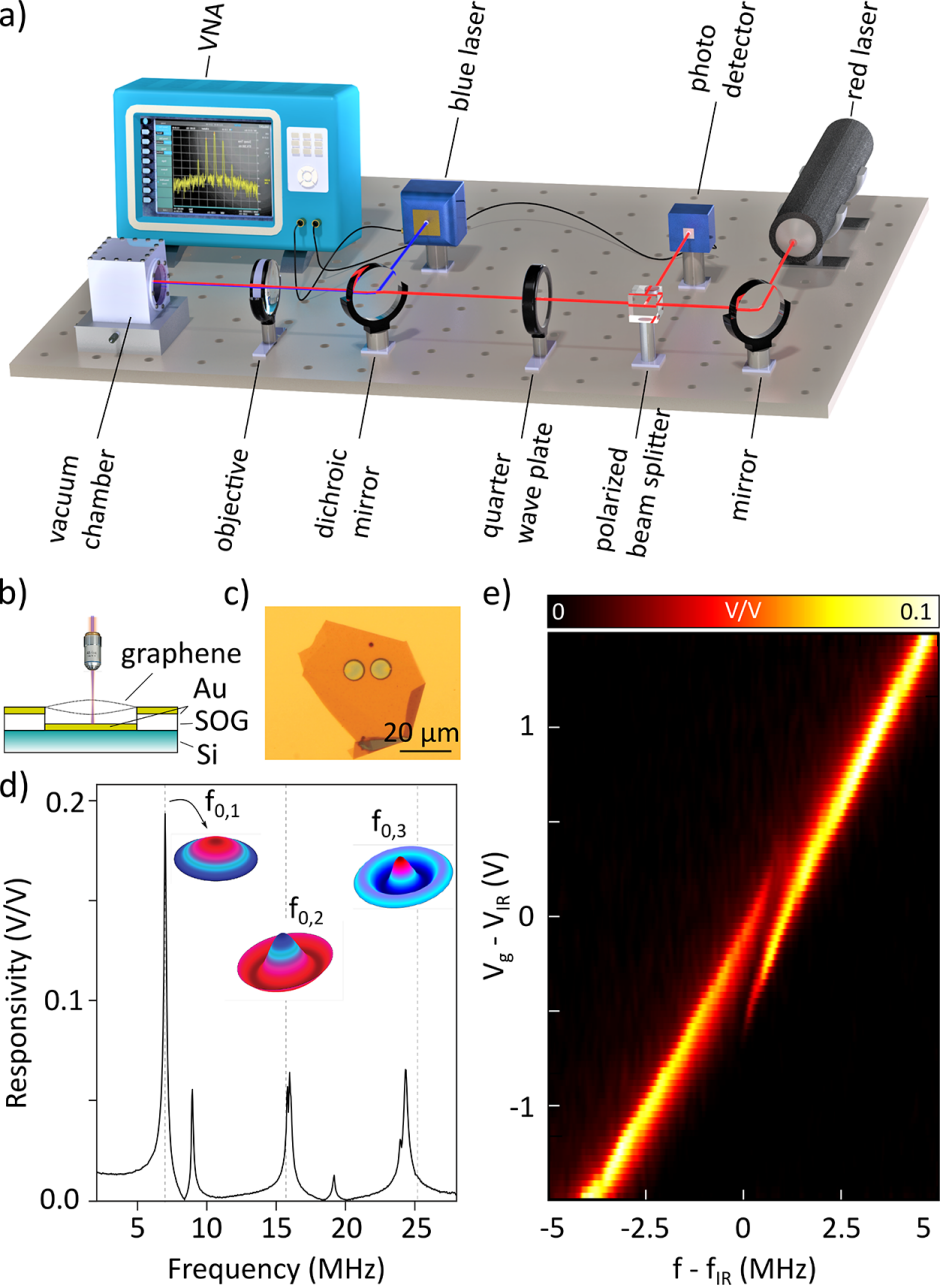
Graphene drum measurements. (a) Schematic of the optical setup for actuating and detecting the motion of graphene. The drum is actuated via a blue laser at a certain frequency set by a VNA, and the motion is read-out using a red laser. (b) Schematic of the resonating graphene drum with electrical contacts. (c) Optical micrograph of the graphene drum. (d) Frequency response of the resonator at neutral gate voltage (*V*_g_ = 0 V). Here, finite element simulations are performed to determine the frequencies of axisymmetric modes of vibration. (e) Variation of the fundamental frequency of the drum *f*_0,1_ as a function of the gate voltage *V*_g_, showing a state of splitting at *V*_IR_ ∼ 7 V and frequency *f*_IR_ = 22.73 MHz.

By sweeping the modulation frequency *f* of the blue laser using a vector network analyzer (VNA), we observe multiple directly driven resonances, appearing as pronounced peaks in the resonator’s spectral response (Figure 1d). Among them, the primary and secondary axisymmetric modes of the drum can be readily identified at *f*_0,1_ = 7.0 MHz (*Q*_0,1_ ≈ 80) and *f*_0,2_ = 15.8 MHz (*Q*_0,2_ ≈ 40) with *f*_0,2_/*f*_0,1_ = 2.25, close to the theoretically predicted ratio of 2.29 for a membrane.^26^ We note that the resonance frequencies depend strongly on the membrane tension, which we can tune via the electrostatic force generated by the electrostatic gate electrode.

By sweeping the gate voltage *V*_g_, we control the tension in the membrane and alter the out of plane offset (see Supporting Information Section 1). The electrostatic force pulls the drum out of its initial flat configuration and breaks its out-of-plane symmetry.^27^ This broken symmetry can have a significant influence on the dynamics of the resonator, especially in the nonlinear regime, where the resonant response deviates from the common Duffing model, because it introduces quadratic terms in the nonlinear stiffness.^28^

We note that increasing *V*_g_ causes the resonance frequencies of the drum to shift at different rates (see Supplementary Figure 1). At a certain critical voltage, *V*_IR_= 7 V, we observe (Figure 1e) splitting of the fundamental resonance peak at *f*_IR_ = 22.73 MHz, which we attribute to the occurrence of a 1:2 internal resonance with a higher mode, since it occurs when the frequency of a higher mode at 44 MHz is exactly twice that of the fundamental mode, see Figure 2a. Besides splitting, the height of both resonance peaks also diminishes close to *V*_IR_, providing evidence for the presence of 1:2 IR and energy redistribution between the interacting modes.

**Figure 2:**
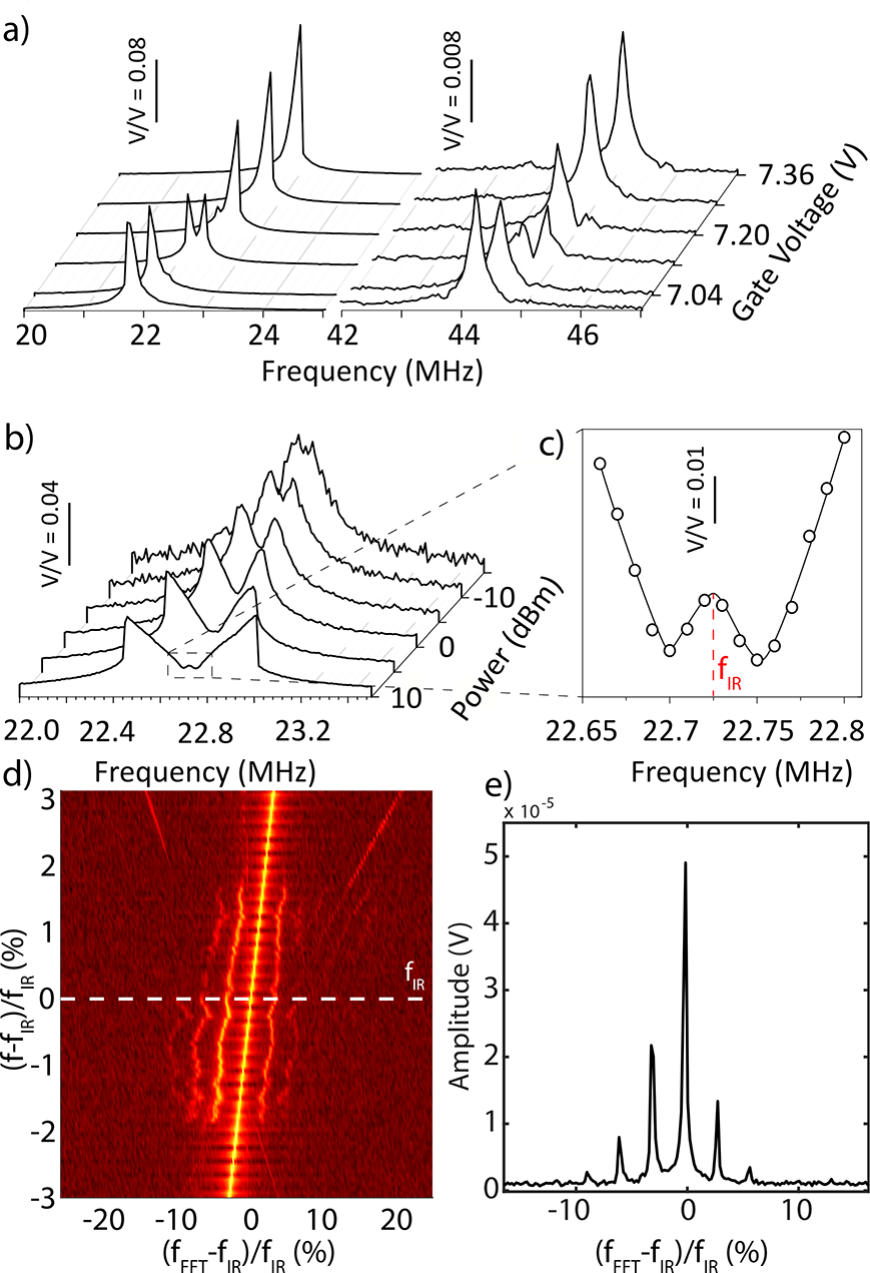
Measured intermodal coupling of the graphene resonator: (a) Evolution of the fundamental and a higher resonance peaks close to the gate voltage of 7.1 V measured via VNA at −10 dBm drive level. (b) Evolution of the 1:2 IR response upon increasing the drive power. (c) A third peak emerges at *f*_IR_. (d) Fast Fourier transform (FFT) measurements at high drive powers while sweeping the blue laser modulation frequency *f*, showing the presence of sidebands at *f*_IR_. The white dashed line in (d) is a line-cute of the FFT map that is zoomed in on (e) to show equally spaced sideband frequencies.

By driving the drum at elevated blue laser powers and performing upward frequency sweeps, we observe in Figure 2b a butterfly-shaped response, consisting of two Duffing-like asymmetric resonances, one of which bending to lower and the other to higher frequency, indicating that one of the split peaks experiences a spring softening, and the other a spring hardening nonlinearity (similar responses have been observed in other nonlinear resonators undergoing IR^29−32^). Interestingly, at the maximum drive level (10 dBm), the strong coupling between the resonant modes yields the emergence of a third peak in the middle of the split region at frequency *f*_IR_ = 22.73 MHz (see Figure 2c).

In order to investigate this unconventional response in depth, we drive the graphene drum to the critical voltage *V*_IR_ required to observe the split peak at *f*_IR_ and use a Zurich UHFLI to analyze the fast oscillations of the drum at high drive powers. By simultaneously tracing the response spectrum while sweeping the driving frequency around *f*_IR_ we noticed that for driving frequencies outside the region where the middle peak was spotted, the motion is harmonic. However, close to *f*_IR_ the spectral response suddenly changes and a frequency comb is observed consisting of multiple equally spaced peaks near *f*_IR_ (see Figure 2d,e and Supporting Information Section 2).

To explain the nonlinear physics associated with the observed dynamics and frequency comb near IR in a system with broken symmetry, we present an analytical model to derive the system’s Lagrangian and obtain the governing equations of motion (see Supporting Information Section 3). For the model, we accounted up to third order nonlinearities, because the graphene drum we study outside the internal resonance regime exhibits a slight Duffing response even at the highest drive levels. We approximate the coupled motion by only considering the drum’s first two axisymmetric modes of vibration with frequencies *f*_0,1_ and *f*_0,2_ (see Figure 1d). For an ideal circular membrane, the ratio of these first two axisymmetric modes can be tuned to approach *f*_0,2_/*f*_0,1_ ≈ 2 by changing the tension distribution. These variations in tension distribution might originate from variations in the electrostatic force if the distance to the gate electrode is nonuniform due to membrane deflection, wrinkling, or buckling. Moreover, to account for the broken-symmetry mechanics, we model the drum with a static deflection from its undeformed state that has the shape of its fundamental mode shape^33^ with an amplitude *W*_0_. This leads to the presence of both quadratic and cubic coupling terms in the equations of motion. However, we note that not all the terms in a 1:2 IR are resonant^9^ and retain only the relevant terms to obtain the following set of simplified equations near the IR (see Supporting Information Section 4)
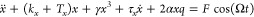
1
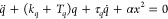
2

Here, *x* and *q* are the generalized coordinates which represent the first and second axisymmetric mode of the graphene membrane respectively, *k*_*x*_ and *k*_*q*_ are the intrinsic mode stiffness, and *T*_*x*_ and *T*_*q*_ represent added stiffness due to electrostatic tuning of the tension. τ_*x*_ and τ_*q*_ are the linear damping coefficients of the generalized coordinates. Moreover, γ is the Duffing coefficient, and α is the coupling strength that can be determined analytically in terms of the offset shape and modes of vibration (see Supporting Information Section 3). Finally, *F* is the forcing amplitude and Ω = 2*πf*_d_ is the excitation frequency. All the terms in eq 1 and eq 2 are mass normalized.

In order to investigate the resonant interaction numerically, we time-integrate the equations of motion. We start by recording the time response of the system at Ω far from resonance and sweep Ω through the 1:2 IR condition. Simulations are performed first at a low driving level that is associated with the linear harmonic oscillator response and then *F* is increased until the specific characteristics of the nonlinear interaction such as mode splitting appear. We perform our simulations using nonlinear parameters γ = 5.78 × 10^30^ (Hz/m)^2^, α = 1.97 × 10^24^ (Hz^2^/m). These values correspond to the analytical model of a 15 nm thick drum with a diameter of 8 μm assuming Young’s modulus of *E* = 1 TPa and initial axisymmetric offset amplitude of 90 nm.

Figure 3a shows the modeled variation of the resonance frequency as a function of the applied tension (*T*_*x*_). By changing the tension *T*_*x*_, the fundamental resonance frequency *f*_0,1_ is tuned and a peak splitting, similar to that in Figure 1e, is observed near the internal resonance frequency *f*_IR_.

**Figure 3:**
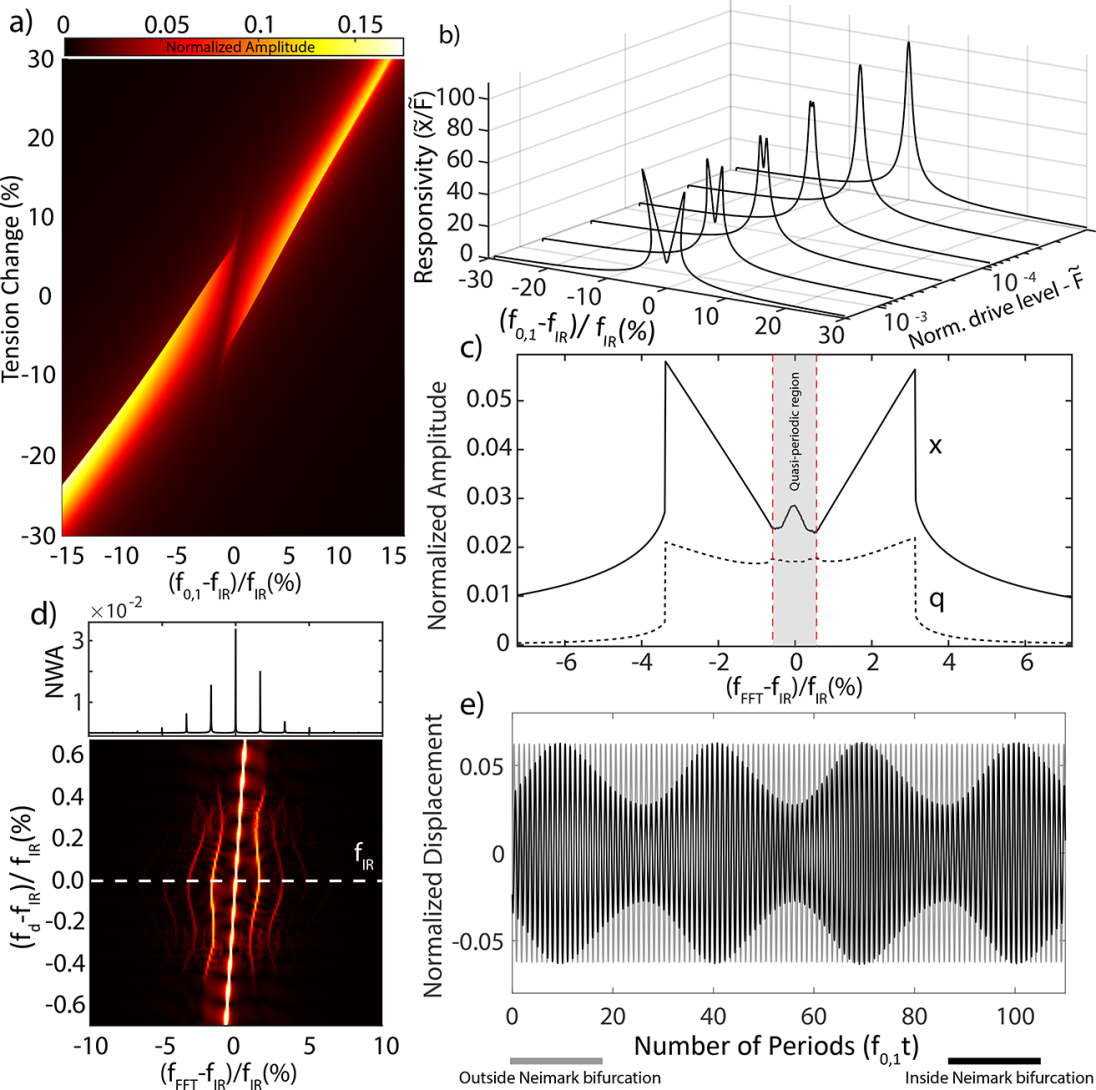
Modal interaction simulations at the normalized drive level *F̃* = *F*/(2*πf*_0,1_*h*) = 0.0015, where *h* is the thickness of the drum. Generalized coordinates are also normalized with respect to the thickness, such that *x̃* = *x*/*h* and *q̃* = *q*/*h*. (a) Frequency response of the fundamental mode as the tension of the membrane is increased. At zero detuning from IR, mode splitting occurs. (b) Frequency response simulations with different drive levels at zero detuning from IR. As the drive level is increased, nonlinear coupling becomes stronger, and both softening and hardening nonlinearities emerge. (c) After a critical drive level, Neimark bifurcations emerge (depicted by red dashed lines) and at the region confined by these bifurcations, the steady-state oscillations become quasi-periodic, generating frequency combs around the resonance frequency. (d) FFT map at the vicinity of IR and critical force level. Frequency combs emerge at the center of the split region, where equally spaced comb elements appear, surrounding the main resonance peak. Inset above is the FFT at the IR condition, showing the normalized wave amplitude (NWA), representing the white dashed line cut of the FFT map. (e) In time domain, this bifurcation leads to amplitude-modulated response.

The splitting phenomenon becomes more apparent at elevated drive powers (see Figure 3b), similar to the experimental observations in Figure 2c. This leads to the emergence of a similar butterfly shaped responsivity *x*/*F*, as the nonlinear coupling becomes stronger at higher drive levels, where energy leaks to the interacting mode. The butterfly shaped split is a direct consequence of the 1:2 IR and can be understood by obtaining the nonlinear frequency response function of eqs 1 and 2 analytically (see Supporting Information Section 3). Interestingly, we also note the presence of the third middle peak in our simulation. In Figure 3c, it can be seen that this peak indeed appears within the split region at zero detuning from IR condition, confirming that 1:2 IR that follows from the equations of motion (1 and 2) can be held accountable for our experimental observations.

In Figure 3c, it can be also noted that when driving near *f*_*IR*_ the second generalized coordinate *q* shows an enhanced amplitude with a response that resembles that of *x*. It is important to note that in the experiments, the middle peak observed at *f*_IR_ is only due to the fundamental amplitude *x*, since our measurements are performed in a homodyne detection scheme.

To better understand the mechanism that lies at the center of our observation, we investigated the stability of the solution branches using a numerical continuation software package (AUTO). We found that the middle peak appears in a region that is confined between two Neimark bifurcations (red dashed lines in Figure 3c and Supporting Information Section 4). Similar to the Hopf bifurcation, at which a fixed point becomes a limit cycle, at a Neimark bifurcation (also known as the secondary Hopf bifurcation) a periodic orbit becomes a quasi-periodic orbit.^34^ Quasi-periodic motion is characterized by a closed invariant curve in the Poincaré map of the phase space that is known to result in amplitude-modulated motion and thus the emergence of frequency combs in the spectral response.^33^

To investigate the spectral characteristics of the quasi-periodic oscillations, we swept the excitation frequency Ω in the spectral neighborhood of the region confined by the two Neimark bifurcations and analyzed the time response of the nonlinear equations, similar to ref (32). Figure 3d shows the frequency content of the simulated time signal inside and outside this region. It can be observed that the frequencies around *f*_IR_ are discretely separated from each other, creating a frequency comb that was nonexistent before reaching the onset of Neimark bifurcation, resembling the frequency comb in Figure 2d. We also show that the time-dependent motion becomes amplitude modulated when entering the Neimark bifurcation region (see Figure 3e), while having constant amplitude outside of that region. Interestingly, numerical simulations also show signatures of chaotic states upon amplification of the drive level, suggesting that 1:2 IR and broken-symmetry mechanics can represent the onset of a transition from quasi-periodic to chaotic oscillations in 2D material resonators (see Figure 4a) and can be tuned by manipulating the intermodal couplings and vibrational states of the drum. However, we note that although the numerical model does capture the most relevant features of the experimental system near the onset of IR, this does not guarantee that this correspondence continues at higher driving levels up to the onset of chaos where other nonlinearities could also play a role. Further experiments will be therefore needed to prove the presented route to chaos near broken-symmetry-induced 1:2 IR.

**Figure 4:**
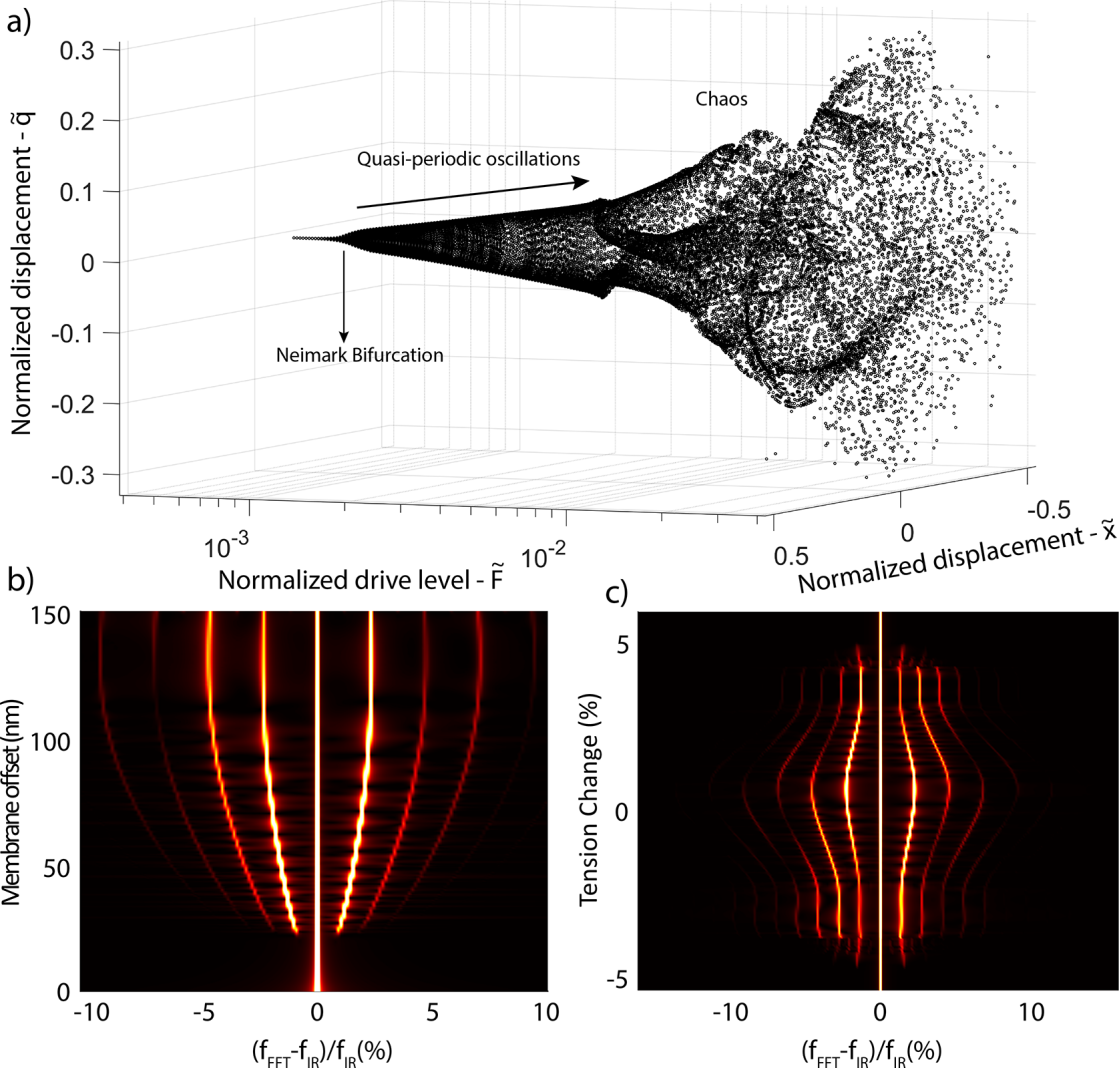
Numerical simulations showing the evolution of phase spaces and sensitivity of frequency comb generation in a graphene drum with broken symmetry and 1:2 IR to (b) offset amplitude (c) tension variation at the drive level *F̃* = 0.0025. (a) Bifurcation diagram of the graphene drum at 1:2 IR, showcasing a quasi-periodic route to chaos. (b) Offset amplitude *W*_0_ has been swept while the FFT of the time signal is being extracted in each step. As the offset increases, so does the boundaries of Neimark bifurcation and comb population. (c) Added stiffness due to the tension change, *T*_*x*_, has been swept while the FFT of the time signal is being extracted in each step, as the added tension moves the resonance frequencies with respect to the 1:2 IR condition.

By simulating the equations of motion at IR while sweeping the parameters, it is also possible to show that the Neimark bifurcations and thus frequency comb generation is sensitive to mechanical parameters of the system. At 1:2 IR, where the Neimark bifurcation is activated, any change in mechanical properties of the drum will be reflected in the frequency spectrum, as a change in the comb intensity, spacing, and population. Figure 4b,c reveals the sensitivity of these combs to the drum offset and tension, which were obtained by sweeping the initial offset (broken symmetry) amplitude and *T*_*x*_. These combs only appear if there is sufficient quadratic nonlinear coupling induced by the broken symmetry, since the terms responsible of the internal resonance are directly related to the membrane offset and diminish in the absence of it (see Supporting Information Section 3). If there is no broken symmetry, the system is symmetric upon inverting the *x* and *q* coordinates and all forces *F*_total_ obey *F*_total_(*x*, *q*) = −*F*_total_(−*x*,–*q*), such that there are only odd (linear and cubic) terms in the equations of motion and therefore no 1:2 interaction and associated combs. Increases in the membrane offset influences both comb spacing and population. Furthermore, near IR, the frequency comb can be used as a sensitive probe for changes in the parameters of the two interacting modes. Any shift in the resonance frequency of the coupled modes results in changes in comb spacings, making it possible to simultaneously probe changes in both frequencies by solely measuring the response of the fundamental mode after the Neimark bifurcation. External parameters like drive power and drive frequency are also observed to influence frequency comb region and serve as controls for tuning comb intensity, spacing, and population (see Supporting Information Section 5).

In summary, we demonstrate a route for generating frequency combs in the nonlinear response of graphene drums that utilizes broken symmetry and 1:2 internal resonance. Unlike other methods that use multiple wave mixing,^35,36^ resonant nonlinear friction,^37^ or SNIC bifurcation^38^ to generate mechanical frequency combs, the presented method makes use of an electrostatic gate to controllably tune frequency combs that are mediated by broken symmetry. When the drum is brought close to the broken-symmetry-induced 1:2 IR, we observe strong splitting of the fundamental resonance peak, exhibiting both softening and hardening nonlinearity. Between the split peaks, we observe resonant interactions when driving at relatively high powers that are generated by Neimark bifurcations of the periodic motion. This regime hosts quasi-periodic oscillations that are held accountable for the observed frequency combs. The experimentally observed phenomena were explained using a continuum mechanics model of a deflected drum with 1:2 IR between its first two axisymmetric modes. Emerging from the inherent geometric nonlinearities, mechanical frequency combs are closely linked to the mechanical properties of 2D materials, including tension, Young’s modulus, and broken symmetry, and thus can be utilized for probing these properties and tracing their variations with frequency and drive levels.^23^ There are many examples in recent years where internal resonance in NEMS/MEMS systems has been utilized to enhance the frequency stability of resonant sensors.^20,39−41^ In these systems, it has also been shown that frequency combs can be used as an alternative approach for resonance frequency tracking.^42^ The internal resonance mechanism described here complements available toolsets in utilizing modal interactions of micro- and nanomechanical systems and paves the way toward controllable use of IR for sensing physical properties of 2D materials and mechanical frequency comb generation.

## Supporting Information Available

Evolution of the overall frequency response with gate voltage, images of membrane displacement under gate voltage, additional experimental results of 1:2 IR and time signal data, derivation of equations of motion for a membrane with initial offset considering two axisymmetric modes, additional simulations of 1:2 IR, frequency response curves, phase spaces and Poincaré maps, drive amplitude, and frequency dependency of frequency combs (PDF)
