# Supplementary material for: Symmetry-Breaking-Induced Frequency Combs in Graphene Resonators
Source: Nano Lett. 2022 Jul 29;22(15):6048–54. doi: 10.1021/acs.nanolett.2c00360 (PMC9373031; doi:10.1021/acs.nanolett.2c00360)
Supplement: Supplementary file 1 — nl2c00360_si_001.pdf [file nl2c00360_si_001.pdf]

Supplementary Information

Symmetry-breaking induced frequency combs in graphene resonators

Ata Keşkekler*[1], Hadi Arjmandi-Tash[1], Peter G. Steeneken[1,2], and Farbod Alijani*[1]

[1]Department of Precision and Microsystems Engineering, Delft University of Technology, Mekelweg 2, Delft 2628 CD, The Netherlands
[2]Kavli Institute of Nanoscience, Delft University of Technology, Lorentzweg 1, Delft 2628 CJ, The Netherlands

July 25, 2022

1 Evolution of the overall frequency response with gate voltage

In Supplementary Figure 1-a (bottom panel) we show the evolution of the resonance frequencies of our graphene drum as a function of the applied gate voltage, and in top panel we show the frequency spectrum at V_g=1.9 V. We observe that change of the gate voltage changes the resonance frequencies at different rates, providing the possibility to obtain IR conditions. $\sim 3\,\mathrm{V}$ is a striking feature of this mapping where there is an abrupt jump in the resonance frequencies. The observation coincides with a rapid change in the observed color of the drum (Supplementary Figure 1-b), which points towards a rapid adjustment in the equilibrium position of the oscillator, i.e. snap-through instability (buckling). This is expected, if the membrane cavity is sealed very well in the atmospheric pressure, where it bulges upwards inside the vacuum chamber due to the pressure difference. This means that center of the membrane is above the surface level of the substrate confirming the presence of initial static deflection. The electrostatic attraction between the membrane and the bottom of the cavity, above the threshold voltage, slowly lowers the center of the membrane, until a critical voltage where an instant jump in the resonance frequencies occurs.

*Corresponding authors: Ata Keşkekler <a.keskekler-1@tudelft.nl>, Farbod Alijani <f.alijani@tudelft.nl>

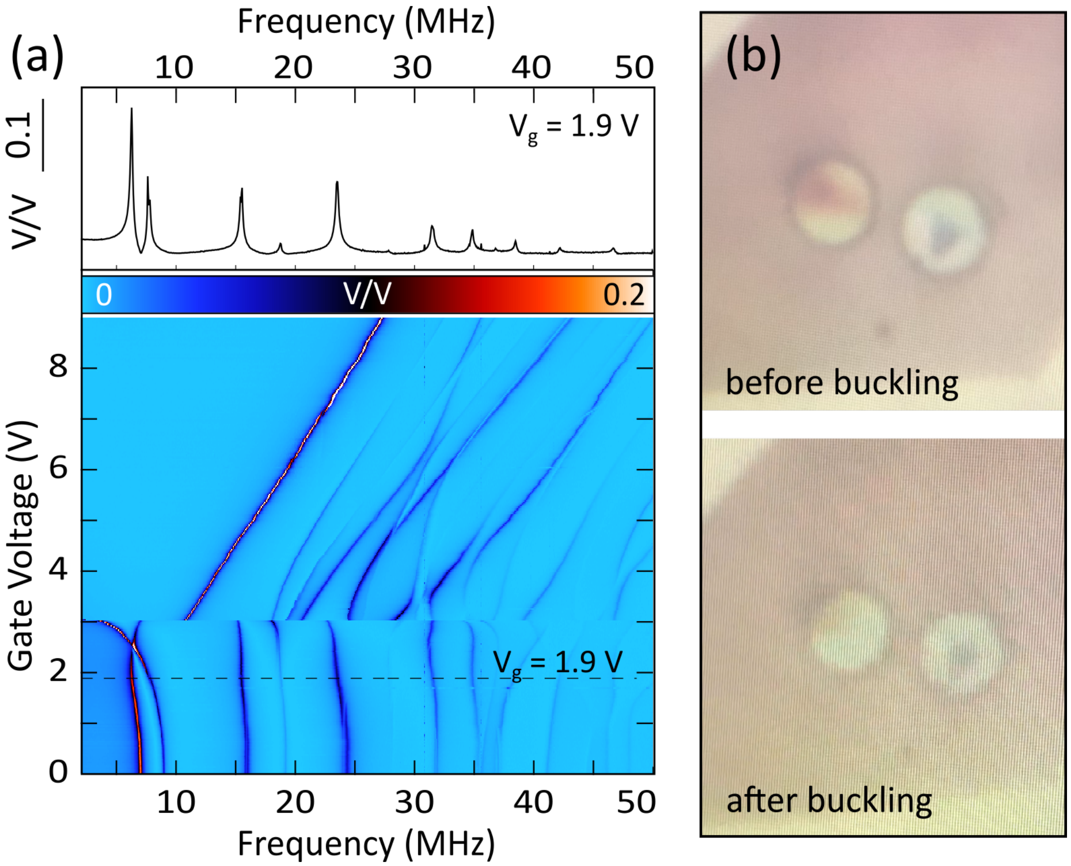

Supplementary Figure 1: Graphene membrane subjected to electrostatic force. (a) Evolution of the resonance frequencies of the membrane as a function of the gate voltage: The resonator showcases several resonance peaks which evolve by applying gate voltage. Note the sudden jump in the resonance frequencies close to the gate voltage of $\sim 3\,\mathrm{V}$ which suggest a state of buckling instability. (b) Snapshots of the membrane under the test (left circular object), before and after the buckling, taken by an optical camera.

2 Additional experimental result

Here, we provide additional experimental result of the same device with different tension and pressure levels, where the 1:2 IR condition was met (Supplementary Figure 2-a). Similar to the main text, we see a nonlinear splitting of the peak as the drive level is increased. Furthermore, in the center of the splitting, the third peak is observed due to Neimark bifurcaitons. The bifurcation gives birth to quasi-periodic motion, which is measured, observed as an amplitude modulated response (see Supplementary Figure 2-b).

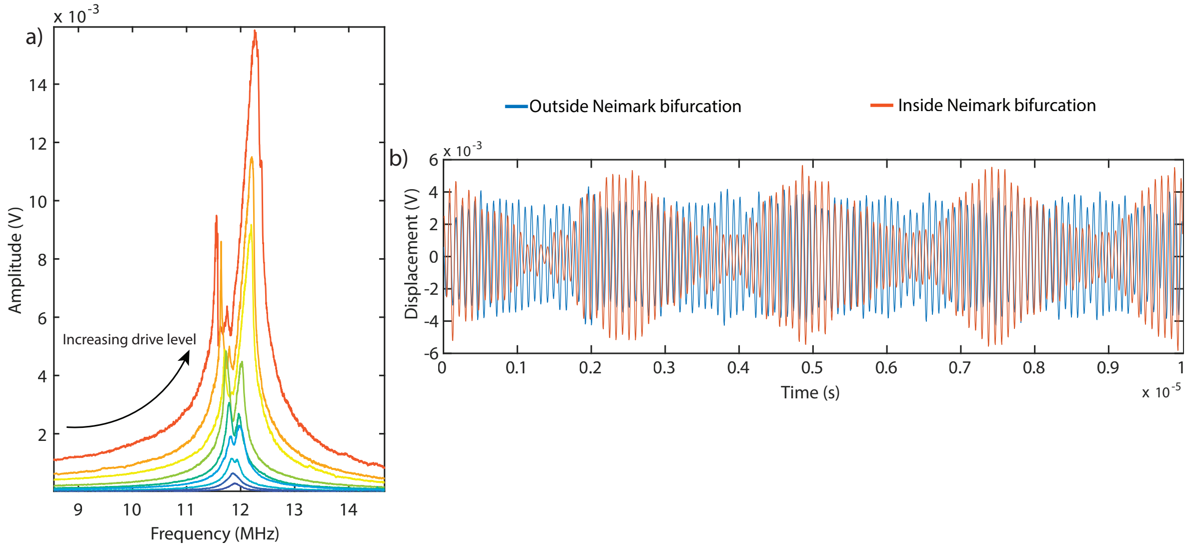

Supplementary Figure 2: Additional experimental results at 1:2 IR. a) As the drive level is increased, the nonlinear splitting occurs. At higher levels, the central peak is also visible. b) Time signal inside and outside the Neimark bifurcation region. Inside the Neimark bifurcation response becomes quasi-periodic, displaying an ampltiude modulation.

3 Equations of motion

To obtain the governing equations of the circular drum, we use the Rayleigh-Ritz approach. The elastic strain energy of the circular drum is obtained as

$$U = \int_0^{2\pi} \int_0^R \frac{Eh}{2(1-\nu^2)} \Big(\epsilon_{rr}^2 + \epsilon_{\theta\theta}^2 + 2\nu\epsilon_{rr}\epsilon_{\theta\theta} + \frac{1-\nu}{2}\gamma_{r\theta}^2 \Big) r dr d\theta, \tag{1}$$

in which h is the thickness of the drum, R is the radius, E is the Young's modulus, and ν is the Poisson's ratio. Moreover, ϵ_{rr}, $\epsilon_{\theta\theta}$, and $\gamma_{r\theta}$ are the normal and shear strains. During axisymmetric oscillations $\gamma_{r\theta} = 0$, and the normal strains are obtained in terms of the transverse deflection (w) and radial displacement (u) of the drum as follows

$$\epsilon_{rr} = \frac{\partial u}{\partial r} + \frac{1}{2}\Big(\frac{\partial w}{\partial r}\Big)^2 + \Big(\frac{\partial w}{\partial r}\Big)\Big(\frac{\partial w_0}{\partial r}\Big), \tag{2}$$

$$\epsilon_{\theta\theta} = \frac{u}{r}. \tag{3}$$

In Eq.(2), w_0 is the initial transverse displacement of the drum associated with zero initial stress. Assuming modal interactions between the first two axisymmetric eigenmodes and fixed boundary conditions ($u = w = 0$), the solution is approximated as

$$w = x(t)J_0\Big(\beta_1 \frac{r}{R}\Big) + q(t)J_0\Big(\beta_2 \frac{r}{R}\Big), \tag{4}$$

$$u = u_0 r + r(R-r)\sum_{j=1}^{n} y_j(t) r^{j-1}, \tag{5}$$

where u_0 is the initial radial displacement due to pre-tension n_0, and x and q are generalized coordinates associated with the first and the second axisymmetric mode of the drum, respectively. Moreover, J_0 is the Bessel function of order zero and β_1 and β_2 are its first two roots. In addition, y_j are the radial generalized coordinates and n is the number of these coordinates retained in the approximation. Moreover, we assume that the initial offset w_0 has the same form of the first axisymmetric mode with known amplitude W_0, thus:

$$w_0 = W_0 J_0\left(\beta_1 \frac{r}{R}\right), \tag{6}$$

The kinetic energy of the drum is then obtained as

$$T = \frac{1}{2}\rho h \int_0^{2\pi} \int_0^{R} \dot{w}^2 r dr d\theta, \tag{7}$$

in which the overdot represents differentiation with respect to time t, and ρ is the mass density. Next, Lagrange equations of motion are obtained [1] leading to the following set of coupled equations

$$\ddot{x} + \omega_1^2 x + \alpha_{11}^{(1)} x^2 + \alpha_{12}^{(1)} xq + \alpha_{22}^{(1)} q^2 + \gamma_{111}^{(1)} x^3 + \gamma_{112}^{(1)} x^2 q + \gamma_{122}^{(1)} xq^2 + \gamma_{222}^{(1)} q^3 = 0, \tag{8}$$

$$\ddot{q} + \omega_2^2 q + \alpha_{11}^{(2)} x^2 + \alpha_{12}^{(2)} xq + \alpha_{22}^{(2)} q^2 + \gamma_{111}^{(2)} x^3 + \gamma_{112}^{(2)} x^2 q + \gamma_{122}^{(2)} xq^2 + \gamma_{222}^{(2)} q^3 = 0, \tag{9}$$

in which $\alpha_{lm}^{(k)}$ and γ_{lm}^{k} are the quadratic and cubic nonlinear terms. It is worth noting that in equations (8) and (9) not all terms are resonant. To recover the resonant terms under 1:2 IR condition ($\omega_2 \simeq 2\omega_1$), we assume harmonic motion of the form $x \approx \cos(\omega_1 t)$ and $q \approx \cos(2\omega_1 t)$ as a first approximation. Inserting these relations in equations 8 and 9 shows that the terms $x^3 \approx \frac{3}{4}\cos(\omega_1 t) + \frac{1}{4}\cos(\omega_1 t)$ and $xq \approx \frac{1}{2}(\cos(\omega_1 t) + \cos(3\omega_1 t))$ in the first equation of motion are trivially resonant. The same holds for the term $x^2 \approx= \frac{1}{2}(1 + \cos(2\omega_1 t))$ which can be viewed as a resonant term in equation 9. In a similar fashion it can be shown that the cubic coupling terms xq^2 and q^2x are dispersive terms. Therefore, the governing equations of motion can reduce to

$$\ddot{x} + \omega_1^2 x + \alpha_{12}^{(1)} xq + \gamma_{111}^{(1)} x^3 = 0, \tag{10a}$$

$$\ddot{q} + \omega_2^2 q + \alpha_{11}^{(2)} x^2 = 0. \tag{10b}$$

Eqs. 10 are the normal form of a coupled oscillator undergoing 1:2 IR, and assuming that the second mode does not surpass its Duffing nonlinearity. It is interesting to note that frequencies ω_1 and ω_2, the coupling term $\alpha_{12}^{(1)} = 2\alpha_{11}^{(2)} = 2\alpha$, and the Duffing nonlinearity $\gamma_{111}^{(1)} = \gamma$ can be expressed in terms of mechanical and geometric properties of the drum as follows

$$\omega_1 = \frac{2.4}{R}\sqrt{\frac{n_0}{\rho h}} \tag{11a}$$

$$\omega_2 = \frac{5.5}{R}\sqrt{\frac{n_0}{\rho h}}, \tag{11b}$$

$$\alpha = 10.794\frac{\pi E h W_0}{R^2}, \tag{11c}$$

$$\gamma = 0.9\frac{\pi E h}{R^2}, \tag{11d}$$

in which α and γ are evaluated assuming $\nu = 0.16$. We note that the Duffing nonlinearity γ depends on the Young's modulus and Poisson's ratio of the drum[1] while the quadratic coupling α in addition depends on the initial deformation w_0. In other words, in the absence of W_0, mechanically, no quadratic coupling exists between the first two eigenmodes of the drum. Moreover, as mentioned above, one can show that in the 1:2 internal resonance scenario, the full model(Supplementary Equations 8 and 9) and the normal form model(Supplementary Equations 10a and b) behave the same qualitatively where they both replicate the observed behavior (Supplementary Figure 3), with only differences in the peak frequencies and the amplitudes due to dispersive terms. Both models display a butterfly shaped response, where left-hand side of the resonance undergoes softening and right-hand side undergoes hardening. This is a direct outcome of 1:2 IR and can be intuitively understood by assuming harmonic motion of the form $x = Xcos(\Omega t)$, $q = Qcos(2\Omega t)$. One can see from Equations (1) and (2) in the main text that

$$[(\omega_1^2 - \Omega^2) + Q\alpha + \frac{3}{4}\gamma X^2]^2 + \tau_x^2 \omega_1^2 X^2 = F^2, \tag{12a}$$

$$Q = \frac{\alpha X^2}{2(4\Omega^2 - \omega_2^2)} \tag{12b}$$

where $\omega_1^2 = k_x + T_x$ and $\omega_2^2 = k_q + T_q$. We note that Supplementary Equations 12a and 12b are approximate solutions that are found by only accounting for the fundamental harmonics and discarding higher order harmonics. By inserting Supplementary Equation 12a in 12b, one can see that the effective nonlinearity is $\gamma_{eff} = \frac{\alpha X^2}{2(4\Omega^2 - \omega_2^2)} + \frac{3}{4}\gamma X^2$. Recalling that close to 1:2 IR, $\omega_2 = 2\omega_1$, for $\Omega < \omega_1$ and by assuming relatively small Duffing constant γ, effective nonlinearity becomes $\gamma_{eff} < 0$ and thus the response is softening at the left side of the resonance, whereas at the right side where $\Omega > \omega_1$, the effective nonlinearity becomes $\gamma_{eff} > 0$, showing a hardening response. This gives rise to the butterfly shaped split observed in the experiments.

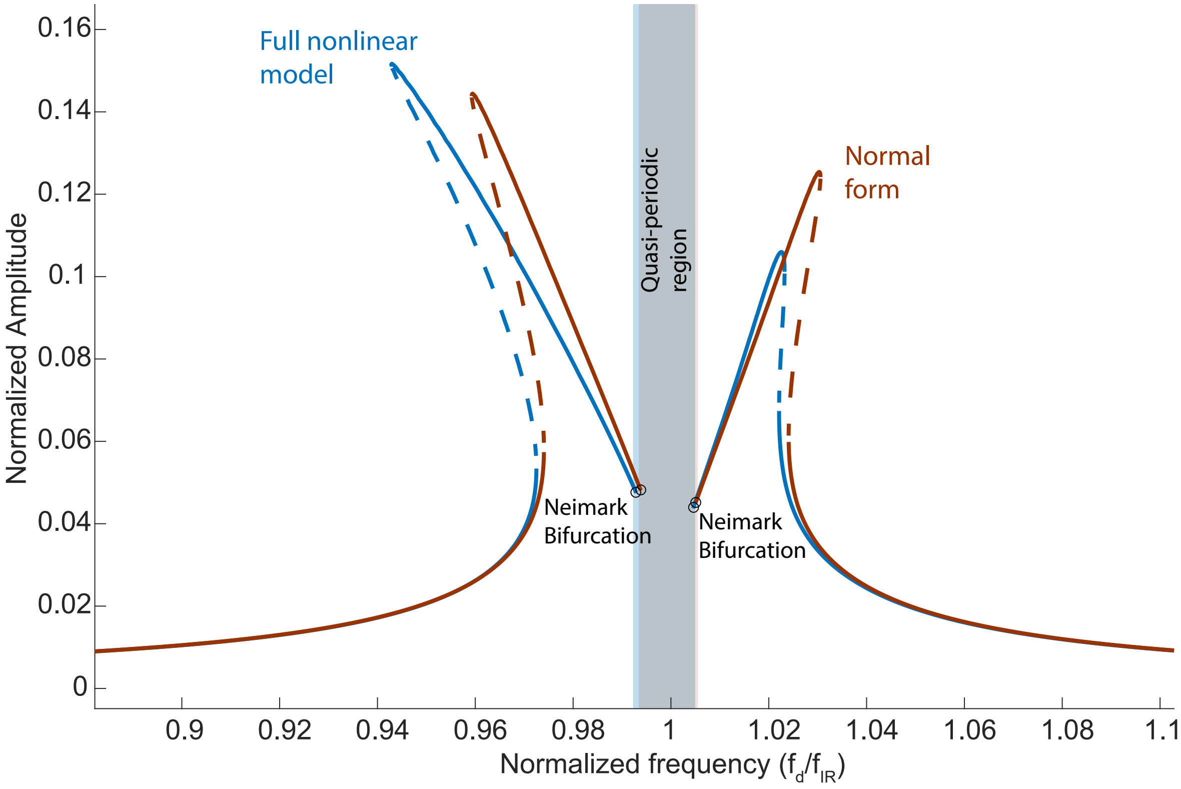

Supplementary Figure 3: Comparison of full model (Supplementary Equations 8 and 9) to normal form model (Supplementary Equation 10). Solid lines depict stable solutions whereas dashed lines depict unstable solutions. Black circles depict the boundaries of the Neimark bifurcation. Physical parameters that are mentioned in the main paper were used in both simulations, with drive $\tilde{F} = 0.002$.

4 Additional simulations

Following on simulations in the main text (Figure 3), here we provide additional simulations. Supplementary Figure 4 shows the frequency response at the internal resonance condition, obtained by the numerical continuation software (AUTO). The Neimark bifurcations are obtained at the points where the motion becomes quasi-periodic in the time integration simulations, also shown in the main text. Furthermore, we investigate the evolution of the phase space as the drive frequency passes the 1:2 IR condition (Supplementary Figure 5). It is possible to observe that, the periodic motion of the resonator turns to quasi-periodic oscillation (which can be also seen from the Poincaré maps) when the bifurcation is triggered (at $f_d/f_{IR} = 0.9951$). The phase space afterwards shows an ergodic behavior till $f_d/f_{IR} = 1.0043$ where the system regains its stable periodic motion through the second Neimark bifurcation. Increasing the drive levels further increases the complexity of the Poincaré maps (Supplementary Figure 6) as the system starts to exhibit chaotic oscillations.

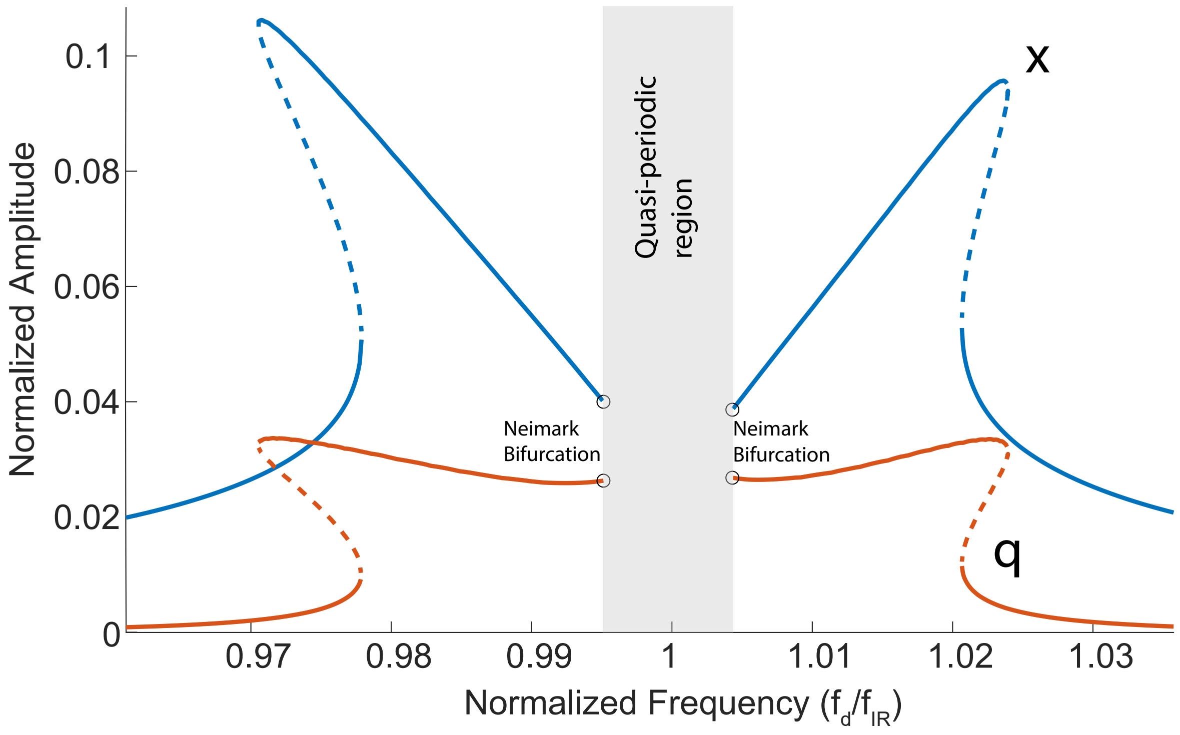

Supplementary Figure 4: Frequency response curves obtained by numerical continuation. Solid lines depict stable solutions whereas dashed lines depict unstable solutions. Black circles depict the boundaries of the Neimark bifurcation.

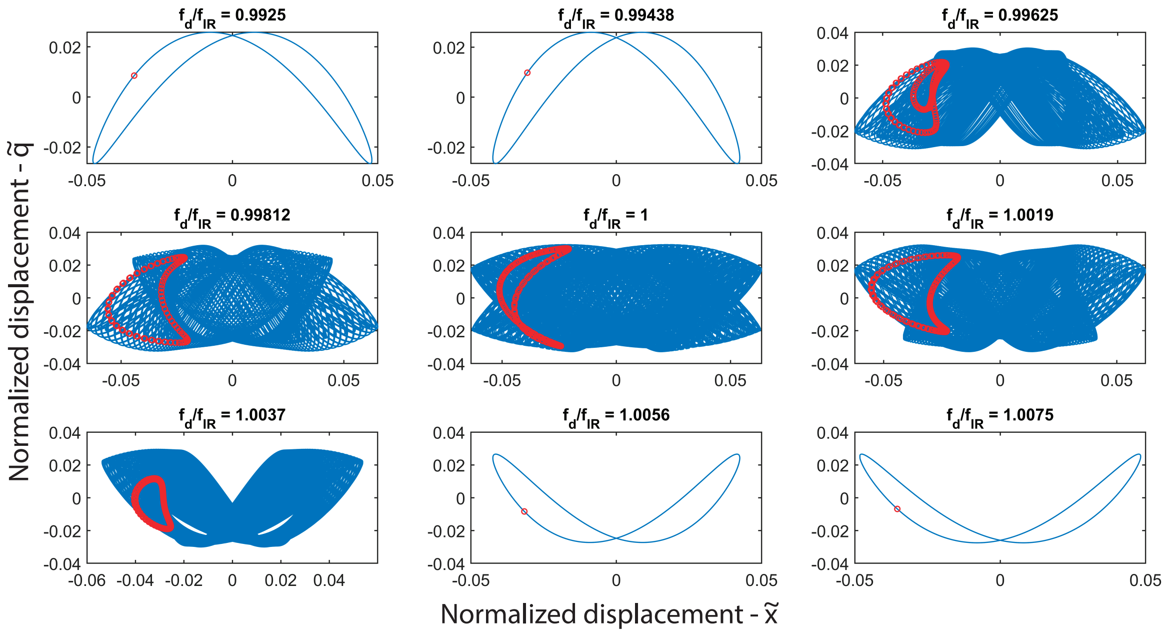

Supplementary Figure 5: Evolution of the phase space as the resonator passes through the IR condition. Poincaré maps are extracted(by taking snapshots at every drive period) on top of the phase spaces, indicated by red circles. ($\tilde{F} = 0.0015$)

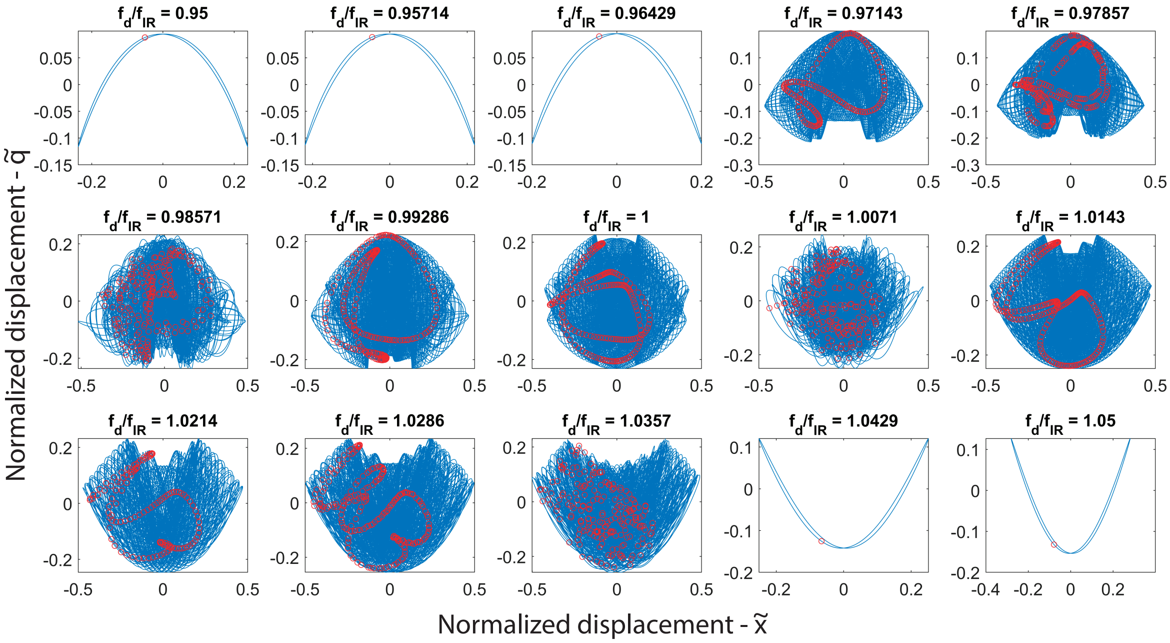

Supplementary Figure 6: Evolution of the phase space at a higher drive level, showing increasingly complex Poincaré maps. ($\tilde{F} = 0.02$)

5 Sensitivity of the frequency combs to the external drive at 1:2 IR

The sensitivity of the comb spacing to the drive frequency can be also studied using our model. In Supplementary Figure 7a it is possible to trace the evolution of the frequency combs as the drive level is increased. Higher drive levels enlarge the Neimark bifurcation region and enrich the spectral response by increasing the comb population, until a certain drive level where the response of the system becomes chaotic. In Supplementary Figure 7b and c we show the variation of comb spacing (Δf) with respect to the drive frequency in the region mediated by Neimark bifurcations, in experiment (from the measurements in Figure 2-d from the main text) and simulations (from the results in Figure 3-d from the main text). It can be seen that the comb spacing is sensitive to the drive frequency.

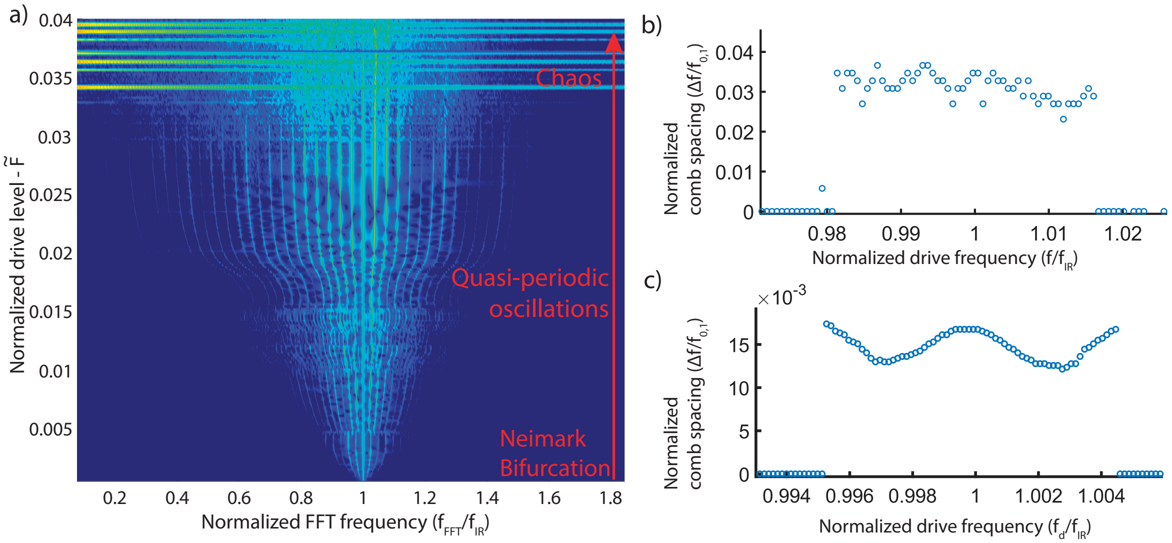

Supplementary Figure 7: (a) As the drive level is increased, the Neimark bifurcation is triggered, giving birth to the frequency comb region. Comb population increases further at high drive levels until the response becomes chaotic. (b) Experimental frequency spacings of the frequency comb as the drive frequency is swept through the 1:2 IR, derived from the results of Fig. 2-d in the main text. (c) Simulated frequency spacings of the frequency comb as the drive frequency is swept through the 1:2 IR, derived from the results of Fig. 3d in the main text.

References

[1] D. Davidovikj, F. Alijani, S. J. Cartamil-Bueno, H. S. Van Der Zant, M. Amabili, and P. G. Steeneken. Nonlinear dynamic characterization of two-dimensional materials. *Nature Communications*, 8(1):1–7, dec 2017.
